# Hospital Housekeeping

**Published:** 1898-08-20

**Authors:** 


### The Institutional Workshop.

### HOSPITAL HOUSEKEEPING.

By a late Matron.

### I.—INTRODUCTORY.

It will hardly be disputed that every hospital matron has had at some period in her career good cause to regret that the teaching of hospital housekeeping is not more technical and definite. Many a nurse or hospital sister is taken straight from ward work and appointed in charge of a cottage hospital or given the matronship of a small country or large provincial hospital, where the housekeeping is mainly, if not entirely, in the hands of the matron and nursing superintendent. Brought face to face—often for the first time—with the problems of Institutional housekeeping, such a matron is apt in her inner consciousness to quake at the new responsibilities put upon her—responsibilities for which her previous training in nine cases out of ten has left her quite unprepared. Outwardly she appears confident, and does her best to grapple with the uncomfortable situation. Nevertheless, realising how little opportunity she has had of organised housekeeping training, she feels distinctly unhappy.

The writer of this article can view the subject from an expert and sympathetic point of view, since it fell to her lot very early in her nursing career to be in just such a catering predicament. Her only preparation had been the training which the average girl receives in a home-life, comprising perhaps a staff of three or four servants, with the added drawback, so far as taking responsibility is concerned, of a mother to advise her in moments of doubt.

What a meagre preparation was this for the housekeeping of an institution of some thirty-five beds, with the entire housekeeping of the patients, the nurses, and the servants to supervise! Yet this is the position in which the average woman finds herself when she receives her first matronship. The writer is fully aware that there are appointments connected with the hospital commissariat known as home sisterships—and that such home sisters take one or two nurses under periodic training in the mysteries and methods of the institutional commissariat. And unquestionably such experience under a home sister is a valuable preparation for a cottage hospital or country matronship. But it is an experience which is not worthy to be called housewifely "training," for it is not sufficiently technical, practical, or definite to be dignified by the title "trained," and, after all, only a few nurses are fortunate enough to gain this experience. To begin with, the home sister under whom the nurse serves her probationary term of housewifery is, rarely enough, herself a trained expert. She has had "experience," and, after some years, is in many ways an admirable housekeeper. But with the real theory and practice of marketing and food supply generally she has only a limited acquaintance. It cannot, therefore, be expected that such hospital housekeepers should have that really expert knowledge which is essential to institutional housekeeping as to every other occupation and profession. Such Sisters have been chosen from the nursing staff for some pre-eminence of practicality or power of organisation of a domestic order. But few, very few, have had a definite training for the work, for the simple reason that so far we possess no school wherein the individual may be trained from the first foundation of institutional housekeeping up to the fine art of catering and marketing on a large scale. In taking counsel together for the improvement and progress of hospital matters generally, we must be prepared to meet criticism, and to candidly confess one to the other wherein the faults of our system lie, and the directions in which improvement may be made. For no reform can come until we open our minds widely, and, freely acknowledging existent faults, lay ourselves out to discover the remedy. So that no reader must misunderstand for one moment the spirit in which I am offering a few suggestions as to the need for the establishment of definite teaching schools in hospital housekeeping and housewifery. At the outset I have much pleasure in testifying to the enormous strides made of late years in the cuisine and commissariat of both hospital patients and nursing staffs. Recalling the unappetising, badly-served, and shockingly cooked meals served in a very important training school in the days of my probation — and this does not represent a time so very long past—I am genuinely impressed with the change that has come over the breakfast and dinner tables of nurses generally. At the same time, having myself had a somewhat expert training in dietetics I cannot say when sitting as a guest at the nurses' boards in institutions, hospitals, &c., that I am satisfied that the present standard has reached a resting point. A great deal yet remains to be done to bring the standard of institutional dieting to a more scientific point. The nurse and the patient are not yet fed with that absolute attention to the food-needs of the worker and of the invalid, as I think should be the case in our times. There is, both in the wards and in the nursing home too much monotony, and a tendency to arrange the meals both of staff and patient by chart.

Beef Monday, mutton Tuesday, with the changes running on five puddings, when fifty others equally nice and equally economical might be occasionally employed with appetising effect. We all know the treadmill feeling engendered in the nursing heart by the knowledge that "to-day being Wednesday we are sure to have stew," and the inevitable same pudding finding its place unvaryingly on the menu which starts with the stew!

I remember some interesting visits paid to an institution in Brooklyn, New York, where a very definite course of teaching could be obtained by anybody desirous of mastering the whole art of the theory and practice of marketing and food supply, and I hope a short account of the method of training may prove equally interesting to my readers. Here, in the three months' course laid down as essential to the training of a hospital housekeeper—either male or female—every possible branch of catering and dietetics was gone into. The students were taught to judge of the food in its uncooked state, to tell by outward and visible signs the exact condition of meat, fish, fowl, and vegetable, and to detect by varying and technical methods with which every person whose duty it is to buy for an institution should be familiar, whether the food offered was up to the standard, and fulfilled the conditions of the hospital contract. Simple tests for adulteration were included in the course, and the whole science of dietetic economy thoroughly and systematically gone into. Periodic visits were made under qualified food escorts to the various city markets, where much valuable and practical information was stored up for future use. In the United States, buying by contract is not the inevitable rule in hospitals. Purchase by contract is, of course, to a large extent a strict necessity to hospital management. In the staple articles of diet, such as bread, milk, and butter, contract supply is not only a convenience, but is, doubtless, more economical in the long run. But the American hospital housekeeper has a good deal of discretionary power in the purchase of meats, fruits, and vegetables in season; for I am glad to record that fruit is rightly considered by American hospital committees to be as important and necessary as is bread in the dieting of both patients and nurses.

